# Protein epigenetic scores and overall mortality in the longitudinal Swedish Adoption/Twin Study of Aging (SATSA)

**DOI:** 10.1186/s13148-025-01843-x

**Published:** 2025-03-05

**Authors:** Thaís Lopes De Oliveira, Arianna March, Jonathan K. L. Mak, Nancy L. Pedersen, Sara Hägg

**Affiliations:** 1 Department of Medical Epidemiology and Biostatistics, Karolinska Institutet, Nobels Väg 12a, 17177 Stockholm, Sweden; 2 Department of Pharmacology and Pharmacy, The University of Hong Kong, Hong Kong SAR, China

**Keywords:** DNA methylation, Epigenetics, Mortality

## Abstract

**Introduction:**

DNA methylation (DNAm) has a functional role in gene regulation, and it has been used to estimate various human characteristics. Variation in DNAm is associated with aging and variability of the proteome. Therefore, understanding the relationship between blood circulating proteins, aging, and mortality is critical to identify disease-causing pathways. We aimed to estimate the association between protein epigenetic scores (EpiScores) and overall mortality in the Swedish Adoption/Twin Study of Aging (SATSA).

**Methods:**

We included information from 374 individuals collected between 1992 and 2014. Our exposures were 109 protein EpiScores generated using DNAm data and prediction models by the MethylDetectR shiny app. All-cause mortality was the outcome of interest. To estimate the protein EpiScores associations with all-cause mortality, we fitted Cox proportional hazard models adjusted for age, sex, education, smoking status, body mass index, and occupation. We also conducted co-twin control analyses to control for shared familial factors.

**Results:**

The mean age of participants at the first assessment was 68.6 years. In total, nine protein EpiScores (e.g., Stanniocalcin 1) were associated with a higher risk for all-cause mortality. In contrast, five protein EpiScores (e.g., Prolyl endopeptidase) were associated with a lower risk for all-cause mortality.

**Conclusion:**

The protein EpiScores associated with an increased mortality risk represent proteins involved in metabolic functions, immune response, and inflammation. Conversely, those associated with a lower risk represent proteins involved in neurogenesis and cellular functions. Overall, it is possible to predict protein levels from DNAm data that could have clinical relevance.

**Supplementary Information:**

The online version contains supplementary material available at 10.1186/s13148-025-01843-x.

## Introduction

Aging is characterized by molecular and cellular changes that may impact an individual’s functional abilities over a lifetime [1]. Among these changes, epigenetic alterations stand out as one of the hallmarks of aging [1]. Epigenetics is a process that modifies gene expression without altering the DNA sequence, yet it can be inherited across generations, e.g., DNA methylation (DNAm) [2].

DNAm is an epigenetic mechanism widely studied in epidemiological research, and it consists of a process where methyl groups are added to the cytosine bases of DNA to form 5-methylcytosine [2]. This mechanism is of particular significance since it regulates gene expression and inhibition [3]. DNAm changes are linked to both aging and environmental factors as well as aging-related diseases and mortality [4–6]. Over time, this biomarker has been used to study biological age acceleration through different epigenetic clocks. Some of these clocks focus solely on chronological age (e.g., Horvath’s epigenetic clock), while others incorporate clinical biomarkers of aging, DNAm-based plasma protein markers, and lifestyle characteristics alongside chronological age (e.g., DNAm PhenoAge and DNAm GrimAge) [5].

In a more recent approach, Gadd and collaborators [7] have developed an epigenetic predictor for protein levels. They generated protein epigenetic scores (EpiScores) using blood DNAm. These EpiScores were associated with the incidence of several common morbidities in aging, such as diabetes and cardiovascular diseases. Proteins serve numerous functions in human physiology to maintain homeostasis [8], acting as structural components within many of the body’s metabolic processes. In aging and aging-related diseases, many molecular and cellular alterations are related to protein abnormalities or altered functionality [1]. Notably, blood proteins have been suggested as potential proteomic aging clocks linked to extending or declining lifespan and health span in animal models [9].

If an epigenetic predictor for protein levels is directly correlated with their corresponding circulating blood proteins, this approach would enable researchers to investigate proteins related to disease-causing pathways with greater reproducibility and reduced susceptibility to transient fluctuations (e.g., acute infections) compared to the corresponding blood proteins. Researchers have examined the relationship between protein EpiScores, diseases, and inflammation [7, 10]; however, to our knowledge, associations with mortality have not been thoroughly evaluated. Therefore, we aimed to estimate the association between protein EpiScores and overall mortality in the Swedish Adoption/Twin Study of Aging (SATSA).

## Methods

### Study population

We used information from the Swedish Adoption/Twin Study of Aging (SATSA). SATSA is a longitudinal study to understand the factors that contribute to healthy aging and longevity. The cohort consists of a large sample of monozygotic (MZ) and dizygotic (DZ) twins (859 individuals) from Sweden. Data collection periods occurred between 1986 and 2014 (Supplementary Fig. S1) [11].

We analyzed data from up to six in-person testing (IPT) occasions (from 1992 to 2014—Supplementary Fig. S1). Each IPT was an extensive data collection that included physical tests, health information, and blood testing [11]. We considered 533 participants with available blood samples with methylation and covariates information. We excluded 159 participants who had missing covariate values (Supplementary Table S1). The final sample size for our main analysis included 374 individuals. For the co-twin control analyses, we excluded occupation as a covariate due to the high proportion of missing values, which would have reduced statistical power (Supplementary Table S1). Consequently, the sample size is larger (*n* = 491, including 176 MZ twins and 315 DZ twins).

### Protein epigenetic scores

DNAm was measured using array technologies (Infinium Human Methylation 450 K Bead Chip [*n* = 385] or Infinium MethylationEPIC BeadChip [*n* = 150], both from Illumina). Information about the methylation process and multi-step quality control pipeline can be found elsewhere [12].

We used the MethylDetectR software [13] to generate the 109 protein EpiScores from blood DNAm data based on Gadd and collaborators work (Table 1) [7]. The authors generated epigenetic scores by training epigenetic data for 953 plasma proteins from the SOMAscan (aptamer-based) and Olink (antibody-based) platforms in two different cohorts (the German population-based study KORA and the Scottish Lothian Birth Cohort 1936) [7]. Of the 953 proteins, 109 met the robustness criteria (*r* > 0.1, *p* < 0.05) and were further validated in the Generation Scotland study. The authors used elastic net penalized regression models to generate the scores. In this model, DNAm at CpG sites, which is predictive of a given protein, was selected by the model, and each selected CpG received a weighting coefficient. The model’s weightings enable MethylDetectR to generate 109 protein EpiScores for our sample using SATSA DNAm data [7, 13].

**Table 1:** List of 109 protein epigenetic scores generated from blood DNA methylation data—SATSA

Protein epigenetic scores
A disintegrin and metalloproteinase with thrombospondin motifs 13 (ADAMTS13)
Activin A receptor like type 1 (ACVRL1)
Adiponectin (ADIPOQ)
Afamin (AFM)
Alpha.L.iduronidase (IDUA)
Alpha-1-antichymotrypsin (SERPINA3)
Aminoacylase 1 (ACY1)
Basal cell adhesion molecule (BCAM)
Beta-2-microglobulin (B2M)
Bone morphogenetic protein 1 (BMP1)
C.X.C motif chemokine 10 (CXCL10—Olink)
C.X.C motif chemokine 10 (CXCL10—SOMAscan)
C.X.C motif chemokine 11 (CXCL11—Olink)
C.X.C motif chemokine 11 (CXCL11—SOMAscan)
C.X.C motif chemokine 9 (CXCL9)
C–C motif chemokine 17 (CCL17)
C–C motif chemokine 18 (CCL18)
C–C motif chemokine 21 (CCL21)
C–C motif chemokine 22 (CCL22)
C–C motif chemokine 25 (CCL25)
CD209 antigen (CD209)
CD48 antigen (CD48)
CD5 antigen-like (CD5L)
Chitotriosidase-1 (CHIT1)
Coagulation factor VII (F7)
Complement C4 (C4A or C4B)
Complement C5a (C5)
Complement C9 (C9)
Contactin.4 (CNTN4)
C-reactive protein (CRP)
C-type lectin domain family 11 member A (CLEC11A—Olink)
C-type lectin domain family 11 member A (CLEC11A—SOMAscan)
C-type mannose receptor 2 (MRC2)
Cytotoxic and regulatory T-cell molecule (CRTAM)
Ectodysplasin A (EDA)
Ectonucleotide pyrophosphatase/phosphodiesterase family member 7 (ENPP7)
Endothelial cell-specific molecule 1 (ESM1)
Eotaxin (CCL11)
Ezrin (EZR)
Fc epsilon receptor II (FCER2)
Fc gamma receptor IIIb (FCGR3B)
Fc receptor like 2 (FCRL2)
Fibroblast growth factor 21 (FGF21)
Galectin 3 binding protein (LGALS3BP)
Galectin.4 (LGALS4)
Granulocyte colony-stimulating factor (CSF3)
Granulysin (GNLY)
Granzyme A (GZMA—Olink)
Granzyme A (GZMA—SOMAscan)
Growth hormone receptor (GHR)
Growth/differentiation factor 8 (GDF8)
Heparin cofactor II (serpin family D member 1, SERPIND1)
Hepatocyte growth factor (HGF)
Hepatocyte growth factor activator (HGFAC)
Hepatocyte growth factor inhibitor (HGFI)
Insulin.receptor (INSR)
Insulin-like growth factor-binding protein 1 (IGFBP1)
Insulin-like growth factor-binding protein 4 (IGFBP4)
Intercellular adhesion molecule 5 (ICAM5)
Interleukin.19 (IL19)
Lactotransferrin (LTF)
Lymphocyte antigen 9 (LY9)
Lymphotoxin beta (LTB)
Lysozyme C
Matrix metallopeptidase 1 (MMP1—Olink)
Matrix metallopeptidase 1 (MMP1—SOMAscan)
Matrix metallopeptidase 12 (MMP12)
Matrix metalloproteinase 2 (MMP2)
Matrix metalloproteinase 9 (MMP9)
Melanoma-derived growth regulatory protein (MIA)
Membrane metalloendopeptidase (MME)
Myeloperoxidase (MPO)
Neural cell adhesion molecule 1 (NCAM1)
Neurotrophic receptor tyrosine kinase 3 (NTRK3—Olink)
Neurotrophic receptor tyrosine kinase 3 (NTRK3—SOMAscan)
Neutral ceramidase (NcDase)
Nicotinamide/nicotinic acid mononucleotide adenylyltransferase 1 (NMNAT1)
Notch receptor 1 (NOTCH1)
Oncostatin-M (OSM)
Osteomodulin (OMD)
Pappalysin.1 (PAPPA)
Platelet glycoprotein Ib alpha chain (GP1BA)
Polymeric immunoglobulin receptor (PIGR)
Prolyl endopeptidase (PREP)
Resistin (RETN)
Retinoic acid receptor responder protein 2 (RARRES2)
S100 calcium binding protein A12 (S100A12)
S100 calcium binding protein A9 (S100A9)
Scavenger receptor cysteine-rich type 1 protein M130 (CD163)
Selectin E (SELE)
Selectin L (SELL)
Semaphorin 3E (SEMA3E)
Serine protease 2 (PRSS2)
Sex hormone-binding globulin (SHBG)
Sialic acid binding Ig like lectin 1 (SIGLEC1)
SLIT and NTRK-like protein 5 (SLITRK5)
SPARC (osteonectin), cwcv and kazal like domains proteoglycan 2 (SPOCK2)
Sphingomyelin phosphodiesterase (SMPD1)
Stanniocalcin 1 (STC1)
T-cell differentiation antigen CD6 (CD6)
Thrombospondin 2 (THBS2)
Thyroid peroxidase (TPO)
Transforming growth factor alpha (TGFA)
Tryptase beta 2 (TPSB2)
Tumor necrosis factor receptor superfamily member 17 (TNFRSF17)
Tumor necrosis factor receptor superfamily member 1B (TNFRSF1B)
Vascular cell adhesion protein 1 (VCAM1)
Vascular endothelial growth factor A (VEGFA)
WAP, Kazal, immunoglobulin, Kunitz and NTR domain-containing protein 2 (WFIKKN2)

Swedish Adoption/Twin Study of Aging—SATSA. The EpiScores were generated using MethylDetectR files

Overall, to generate the EpiScores using MethylDetectR software, the DNAm data, in beta values format, should be uploaded into the software. The software imputes missing beta values (mean imputation) and assigns missing CpG sites the mean beta value from Generation Scotland DNAm data. It is also possible to locally generate the protein EpiScores (using the same imputation methods) with the R codes and data files provided by MethylDetectR [13]. We chose to generate our EpiScores locally. (The data was not uploaded to MethylDetectR software.)

It is also worth mentioning that the protein EpiScores are returned without a unit/scale; however, they correlate with their target protein in the sense that those on the lower end of the scale should have lower protein values than those at the higher end [7, 13]. Hence, we standardized our scores using z-score standardization (mean value is 0, and the standard deviation is 1).

### Overall mortality

All-cause mortality information (vital status and dates of death) was derived from the Swedish National Death Registry. The dates of death were updated on 01 October 2022 (date of censuring).

### Time-stable and time-varying confounders

The time-stable confounders were sex, education, and occupation. Sex was treated as 1 = men and 2 = women. Education was categorized as 1 = elementary school, 2 = 0-level vocational school or folk high school, 3 = gymnasium, and 4 = university or higher. Occupation was defined as the work or profession the participants have had for most of their working life. The categories included: 1 = unskilled and semiskilled workers, 2 = skilled workers, 3 = assistant non-manual employees, 4 = intermediate non-manual employees, 5 = employed and self-employed professionals (higher civil servants and executives), 6 = self-employed (other than professionals), 7 = housewife (or male equivalent), and 8 = housewife with a temporary job. Education and occupation were considered a time-stable confounder because they are unlikely to change after 50 years of age.

The time-varying confounders included in our study were age (time scale), smoking status, and body mass index (BMI). All time-varying confounders were measured on the same date as the blood sample collection. Smoking status was treated as 1 = not currently smoking, 2 = ex-smoker, and 3 = currently smoking. BMI was numeric and calculated as kilograms divided by squared height in meters.

### Statistical analyses

Means, standard deviations, proportions, and frequency were used to describe characteristics of the study population regarding the protein EpiScores, overall mortality, and confounders. Descriptive Pearson correlation plots between the protein EpiScores and age are included in Supplementary Figs. S2, S3, and S4.

The effects of the 109 protein EpiScores on overall mortality were estimated through Cox proportional hazard models. First, to create the time scale, we considered the earliest chronological age, in months, as the initial time, and from this age, the entry time of each individual was calculated. We utilized a counting process to account for time-varying confounders. In this time-varying analysis, all variables were included at each time point [14].

We performed a separate model for each of the 109 protein EpiScores. We considered hazard ratios (HR) with a 95% Confidence Interval (CI) as significant; however, we only interpreted HR that were significant after adjustments for multiple comparisons (False Discovery Rate, FDR). Model 1 refers to estimated crude HR adjusted for age (time scale) and sex. Model 2 refers to estimated HR adjusted for age (time scale), sex, education, smoking status, BMI, and occupation. In all models, we considered the relatedness of the twin pairs. The accumulated risks of overall mortality for the protein EpiScores during the entire study period are shown in Supplementary Fig. S5 and S6. Additionally, we evaluated whether the risk factor protein EpiScores were correlated with one another (Supplementary Fig. S7). A similar assessment was conducted for the protective factor protein EpiScores (Supplementary Fig. S8). Finally, we conducted residuals analysis with different purposes. We verified Schoenfeld residuals to check the assumption of proportional hazards, Deviance residuals to check outliers, and Score residuals to check influential or extreme observations. Lastly, Harrell’s C-index was described in the models to check the global assessment of discrimination. Values close to one have better discriminatory power.

To evaluate the familial and shared environmental influences, we performed stratified Cox proportional hazard models within MZ and DZ twin pairs. In these analyses, model 2 was not adjusted for occupation. We also tested the correlation of protein EpiScores values within MZ twin pairs as sensitivity analysis. All analyses were conducted using R software [15], version 4.3.1, package tidyverse, heatmaply, and survival.

## Results

In the initial assessment, the mean age of participants was 68.6 years. Most of the study population consisted of women (61%), 50.5% completed elementary school, and 29.1% were unskilled and semiskilled workers. Additionally, 80.5% were non-smokers, with a mean body mass index of 26 in the initial assessment. These proportions remained consistent throughout the entire study period. The average follow-up time was 16 years and further two IPTs per individual. The number of participants who had died was 253 (Table 2). The 109 protein EpiScores exhibited a range from approximately −8.5 to 7.2 (Supplementary Table S2). Furthermore, these protein EpiScores had weak correlations with age, with values varying between −0.4 and 0.4 (Supplementary Figs. S2, S3, and S4).

**Table 2:** Descriptives of study variables in the first assessment and in the whole study period, SATSA

	First assessment (*n* = 374)	Whole period (all measurements, *n* = 947)
Age (years)—Mean (SD)	68.6 (9.8)	71.9 (9. 4)
*Sex—n (%)*		
Men	146 (39.0%)	376 (39.7%)
Women	228 (61.0%)	571 (60.3%)
*Education—n (%)*		
Elementary school	189 (50.5%)	459 (48.5%)
0-level of vocational school or folk high school	123 (32.9%)	321 (33.9%)
Gymnasium	27 (7.2%)	71 (7.5%)
University or higher	35 (9.4%)	96 (10.1%)
*Socio-economic classification*		
Unskilled and semiskilled workers	109 (29.1%)	278 (29.4%)
Skilled workers	37 (9.9%)	101 (10.7%)
Assistant non-manual employees	71 (19.0%)	198 (20.9%)
Intermediate non-manual employees	56 (15.0%)	144 (15.2%)
Employed and self-employed professionals (higher civil servants and executives)	23 (6.1%)	64 (6.8%)
Self-employed (other than professionals)	21 (5.6%)	43 (4.5%)
Housewife (or male equivalent)	51 (13.6%)	101 (10.7%)
Housewife with a temporary job	6 (1.6%)	18 (1.9%)
*Smoking status—n (%)*		
Current smoker	62 (16.6%)	136 (14.4%)
Ex-smoker	11 (2.9%)	28 (3.0%)
Non-smoker	301 (80.5%)	783 (82.7%)
Body Mass Index (kg/m^2^)—Mean (SD)	26.0 (4.1)	26.3 (4.2)
Follow-up time (years)—Mean (SD)	–	16.9 (8.0)
In-person tests—Mean (SD)	–	2.1 (1.1)

SATSA, Swedish Adoption/Twin Study of Aging. Age (time scale), smoking status and body mass index were time-varying covariates

### Protein EpiScore and overall mortality

In Cox proportional hazard models (after multiple comparisons correction), the increase of one standard deviation of IGFBP4, MMP9, OSM, PIGR, RARRES2, S100A12, STC1, TGFA, and THBS2 was associated with an increased risk of overall mortality: HR of 1.23, 1.23, 1.32, 1.31, 1.21, 1.21, 1.48, 1.31, and 1.18, respectively (Table 3). MMP9, OSM, PIGR, S100A12, and TGFA exhibited strong correlations with each other (*r* > 0.70) (Supplementary Fig. S7). Conversely, the increase of one standard deviation of LY9, NCAM1, PREP, SEMA3E, and WFIKKN2 were associated with a lower risk of overall mortality: HR of 0.80, 0.78, 0.83, 0.79, and 0.80, respectively (Table 3). LY9, NCAM1, and SEMA3E exhibited moderate correlation with each other (*r* > 0.50) (Supplementary Fig. S8). The estimated concordance probability of all models had a predictive value ranging from 64 to 66% (Harrell’s C-index). The hazard ratios for the 109 protein EpiScores are detailed in Supplementary Table S3. Throughout the study period, the cumulative risk of overall mortality progressively increased, particularly toward the end, for all protein EpiScores (Supplementary Figs. S5 and S6).

**Table 3:** Crude and adjusted associations between protein EpiScores and overall mortality in SATSA

	Model 1- crude models (*n* = 374)		Model 2- adjusted models (*n* = 374)		
	Hazard ratio* (95% CI)	Harrell’s C-index	Hazard ratio* (95% CI)	Harrell’s C-index	Corrected *p*-value
*Protein EpiScores considered risk factor*					
IGFBP4	1.25 (1.10–1.43)	0.61	1.23 (1.07–1.41)	0.65	0.03
MMP9	1.34 (1.17–1.52)	0.64	1.23 (1.06–1.42)	0.66	0.05
OSM	1.40 (1.23–1.59)	0.62	1.32 (1.14–1.51)	0.65	< 0.01
PIGR	1.41 (1.23–1.63)	0.64	1.31 (1.10–1.56)	0.66	0.03
RARRES2	1.20 (1.06–1.35)	0.61	1.21 (1.07–1.38)	0.65	0.03
S100A12	1.24 (1.11–1.39)	0.60	1.21 (1.07–1.36)	0.64	0.03
STC1	1.43 (1.17–1.73)	0.61	1.48 (1.22–1.80)	0.65	< 0.01
TGFA	1.40 (1.23–1.60)	0.64	1.31 (1.14–1.51)	0.66	< 0.01
THBS2	1.22 (1.08–1.38)	0.61	1.18 (1.05–1.34)	0.66	0.05
*Protein EpiScores considered protective factor*					
LY9	0.79 (0.69–0.92)	0.61	0.80 (0.70–0.93)	0.65	0.03
NCAM1	0.77 (0.67–0.87)	0.62	0.78 (0.67–0.90)	0.65	0.03
PREP	0.86 (0.76–0.98)	0.60	0.83 (0.73–0.94)	0.64	0.03
SEMA3E	0.74 (0.64–0.86)	0.62	0.79 (0.67–0.94)	0.65	0.05
WFIKKN2	0.76 (0.67–0.87)	0.62	0.80 (0.70–0.92)	0.65	0.03

This table includes only proteins EpiScores with significant estimates in Cox proportional hazard models after FDR correction (corrected *p*-value). The Supplementary Table S3 shows the hazards ratios for all 109 protein EpiScores. SATSA: Swedish Adoption/Twin Study of Aging. Model 1 were adjusted for age (time scale) and sex. Model 2 were adjusted age (time scale), sex, education, smoking, body mass index, and occupation

95% CI, Confidence interval 95%; IGFBP4, Insulin-like growth factor-binding protein 4; LY9, Lymphocyte antigen 9; MMP9, Matrix metalloproteinase 9; NCAM1, Neural cell adhesion molecule 1; OSM, Oncostatin-M; PIGR, Polymeric immunoglobulin receptor; PREP, Prolyl endopeptidase; RARRES2, Retinoic acid receptor responder protein 2; S100A12, S100 calcium binding protein A12; SEMA3E, Semaphorin 3E; STC1, Stanniocalcin 1; TGFA, Transforming growth factor alpha; THBS2, Thrombospondin 2; WFIKKN2, WAP, Kazal, immunoglobulin, Kunitz and NTR domain-containing protein 2

The residual analyses revealed that NTRK3—Olink violated Schoenfeld’s assumption (Supplementary Table S4 and Supplementary Fig. S9). Hence, despite having significant estimates in the Cox proportional hazard models, its estimates were not interpreted. Additionally, we observed a few outliers (deviance residuals—Supplementary Table S5) but no influential or extreme observations (score residuals—Supplementary Table S6).

**Fig. 1:**
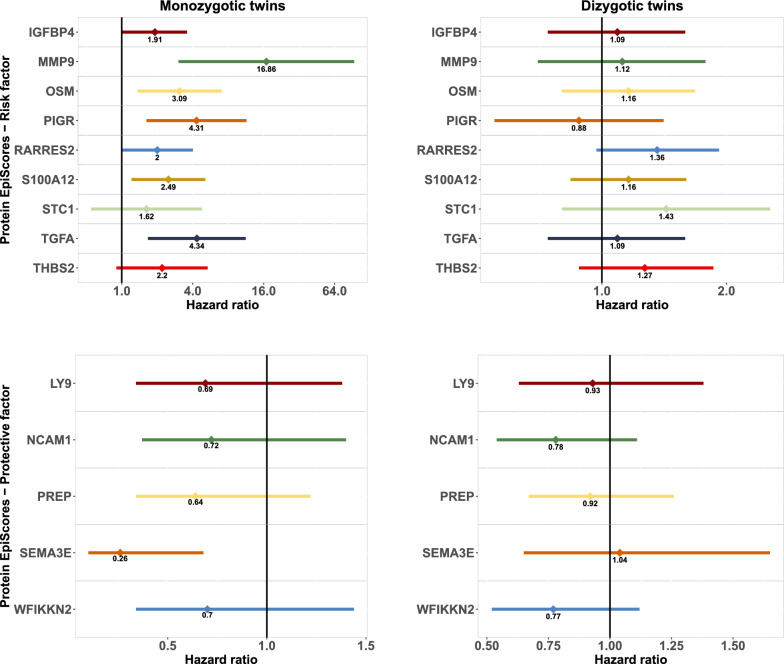
Forest plot with Cox proportional hazards from co-twin control analyses, SATSA (*n* = 491 individuals). Note: The values represent hazard ratios (HR) with a 95% Confidence Interval (CI) from stratified Cox proportional hazard models within MZ and DZ twin pairs. Swedish Adoption/Twin Study of Aging—SATSA. The confidence interval for MMP9 ranges from 3.03 to 93.7; however, it was not represented in the figure to enhance visualization. IGFBP4: Insulin-like growth factor-binding protein 4, LY9: Lymphocyte antigen 9, MMP9: Matrix metalloproteinase 9, NCAM1: Neural cell adhesion molecule 1, OSM: Oncostatin-M, PIGR: Polymeric immunoglobulin receptor, PREP: Prolyl endopeptidase, RARRES2: Retinoic acid receptor responder protein 2, S100A12: S100 calcium binding protein A12, SEMA3E: Semaphorin 3E, STC1: Stanniocalcin 1, TGFA: Transforming growth factor alpha, THBS2: Thrombospondin 2, WFIKKN2: WAP, Kazal, immunoglobulin, Kunitz and NTR domain-containing protein 2

In Cox proportional hazard models within twin pairs, the HRs for the protein EpiScores were not statistically significant for DZ twin pairs. In contrast, for MZ twin pairs, we observed stronger counterintuitive associations with substantial confidence intervals (Fig. 1 and Supplementary Table S7). In our sensitivity analysis (protein EpiScores correlation within MZ twin pairs, Supplementary Fig. S10), we observed that most protein EpiScores had poor or moderate correlation within these twin pairs. This suggests a potentially lower genetic correlation. However, STC1 was an exception, showing a correlation higher than 0.7 (Supplementary Fig. S10).

## Discussion

In this study, we investigated the association between protein EpiScores and overall mortality. We found nine protein EpiScores associated with an increased overall mortality risk and five EpiScores associated with a decreased overall mortality risk after adjustments for age (time scale), sex, education, smoking status, BMI, and occupation.

The protein EpiScores associated with an increased mortality risk represent proteins involved in metabolic functions, immune response, and inflammation. Additionally, some represent proteins acting as growth factors/promoters. Specifically, IGFBP4, RARRES2, S100A12, and STC1 have metabolic functions, such as renal phosphate reabsorption and regulation of inflammatory processes [7, 16–19]. MMP9, OSM, PIGR, and THBS2 play crucial roles in the immune response and inflammation. These roles include, for example, facilitating cell-to-cell interactions, transporting antibodies, and regulating the growth and differentiation of various cell types [20–23]. Lastly, TGFA regulate cell growth, such as angiogenesis and tissue regeneration [24]. Most of these proteins are related to the pathogenesis of numerous diseases, such as cancers and cardiovascular diseases [16, 17, 20, 24]. In previous research, these protein EpiScores were also associated with the incidence of ischemic heart disease, chronic obstructive pulmonary disease, lung cancer, depression, and diabetes [7].

It is worth noticing that these proteins interact with each other, and a high level of a protein EpiScore might not necessarily indicate they have a role in diseases or mortality causal paths. As an example, Gude et al. describe the relationship between PAPPA, IGFBP1, IGFBP4, and STC1 in cardiovascular disease [25]. In this scenario, STC1 may be elevated as a compensatory mechanism in response to elevated levels of PAPPA, despite its association with a high risk of all-cause mortality. Hence, it is essential to conduct additional studies to explore the potential role of these proteins or protein EpiScores in disease causal pathways through stronger causal inference methods [26].

The protein EpiScores associated with a lower mortality risk represent proteins acting as growth factors and involved in inflammation, immune response, neurogenesis, and cellular functions. Specifically, LY9 and SEMA3E have significant roles in immune response, including the regulation of various immune cell activities and functions [27, 28]. NCAM1 is a protein regulating neurogenesis, neuron-neuron adhesion, neurite fasciculation, outgrowth of neurites, and cell migration [29]. PREP is a cell surface glycoprotein serine protease that participates in many cellular processes in tissue remodeling and inflammation [30]. Lastly, WFIKKN2 inhibits the biological activity of mature myostatin [7]. In previous research, PREP and WFIKKN2 EpiScores were considered protective factors to incident chronic obstructive pulmonary disease and stroke, respectively [7].

Limited research is found concerning associations between mortality and the proteins corresponding to the EpiScores identified here [25, 31–38]. The studies are often conducted with small samples or in specific settings, e.g., institutionalized participants or participants from clinics, and populations with specific conditions, e.g., participants in hemodialysis. However, our results in community-dwelling older twin adults appear to be robust and in the same direction of association as previous research. To the best of our knowledge, our study was the first to describe associations between the protein EpiScores and overall mortality.

We also conducted a co-twin control analysis to estimate the association between the protein EpiScores and overall mortality, considering the interplay between environmental factors and genetics [39]. However, we could not extract much information from the co-twin control analysis due to limited statistical power (most associations were non-significant). In a sensitivity analysis, we found that the EpiScore STC1 exhibited the highest correlation among MZ twins, suggesting a potentially stronger genetic influence despite its non-significant association in the within-pair analysis. The other EpiScores showed weaker or moderate correlations within the MZ twins. It is reasonable to conclude that the protein EpiScores may be influenced by environmental characteristics since they are created using an epigenetic mechanism (DNAm), and external factors, such as drugs, nutrition, and chemicals, may influence epigenetics alterations [40]. This influence of the environmental characteristics on DNAm is a limitation of this study.

Another limitation is our sample size. Although we had longitudinal data to estimate the associations between protein EpiScores and overall mortality over time, we could not perform stratified analysis by sex due to a lack of statistical power. Additionally, we were unable to consider important confounders in our analysis, such as race/ethnicity. It is well-established that men and women differ in many biological aging traits [41], and that ethnic differences exist. Therefore, we recommend that future research further analyze sex and ethnic differences concerning the protein EpiScores. Furthermore, as in any longitudinal study, attrition occurred in SATSA, resulting in individuals remaining with better health status. Hence, the generalizability of our study to the general population must be considered with caution. Finally, SATSA did not measure the corresponding blood protein values, preventing us from providing correlations between EpiScores and their corresponding blood proteins as a validation metric.

To conclude, we assessed the protein EpiScores developed by Gadd and collaborators in a distinct cohort and found that 14 protein EpiScores were significantly associated with overall mortality. This study discussed the functionality of the blood proteins associated with these significant EpiScores and addressed their relevance in different health outcomes. Overall, it is possible to predict protein levels from DNAm data, which could have clinical relevance.

## Supplementary Information

Additional file 1.

## Data Availability

The data cannot be shared publicly by the authors. The Swedish Adoption/Twin Study of Aging (SATSA) data is available from the National Archive of Computerized Data on Aging (NACDA) 2015. https://www.icpsr.umich.edu/web/NACDA/studies/3843/summary.
