# Supplementary material for: Protein epigenetic scores and overall mortality in the longitudinal Swedish Adoption/Twin Study of Aging (SATSA)
Source: Clin Epigenetics. 2025 Mar 5;17:41. doi: 10.1186/s13148-025-01843-x (PMC11881402; doi:10.1186/s13148-025-01843-x)
Supplement: Supplementary file 1 — Additional file 1. [file 13148_2025_1843_MOESM1_ESM.docx]

Supplementary Fig. S1 - Data collection periods of the Swedish Adoption/Twin Study of Aging - SATSA.


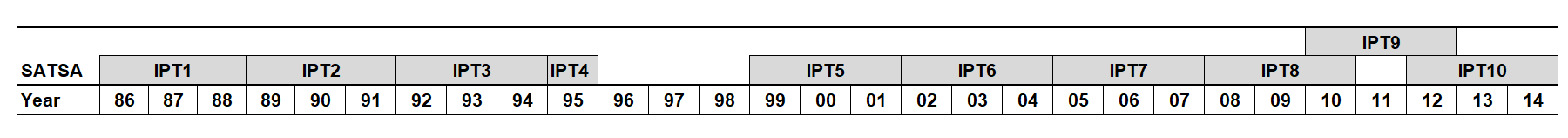


Note: The in-person data collection occurred between 1986 and 2014. In this study, we used information from IPT3, IPT5, IPT6, IPT8, IPT9, and IPT10.

Supplementary Table S1- First assessment descriptive information on missing data in the Swedish Adoption/Twin Study of Aging - SATSA (n= 159 individuals).

|  | First assessment (n= 159) |
| --- | --- |
| **Age - Mean (SD)** | 68.9 (9.2) |
| **Sex – n (%)** |  |
| Women | 86 (54.1%) |
| Men | 73 (45.9%) |
| **Education – n (%)** |  |
| Elementary school | 94 (59.1%) |
| 0-level of vocational school or folk high school | 32 (20.1%) |
| Gymnasium | 10 (6.3%) |
| University or higher | 4 (2.5%) |
| Missing | 19 (11.9%) |
| **Occupation** |  |
| Unskilled and semiskilled workers | 4 (2.5%) |
| Skilled workers | 3 (1.9%) |
| Assistant non-manual employees | 1 (0.6%) |
| Intermediate non-manual employees | 2 (1.3%) |
| Employed and self-employed professionals (higher civil servants and executives) | 1 (0.6%) |
| Self-employed (other than professionals) | 1 (0.6%) |
| Housewife (or male equivalent) | 4 (2.5%) |
| Housewife with a temporary job | 0 (0%) |
| Missing | 143 (89.9%) |
| **Smoking status – n (%)** |  |
| Current smoker | 29 (18.2%) |
| Ex-smoker | 6 (3.8%) |
| Non-smoker | 95 (59.7%) |
| Missing | 29 (18.2%) |
| **Body Mass Index - Mean (SD)** | 26.0 (3.58) |
| Missing – n (%) | 30 (18.9%) |

Supplementary Fig. S2- Pearson correlation plots of the 109 protein EpiScores over age in the Swedish Adoption/Twin Study of Aging - SATSA (n= 374 individuals).


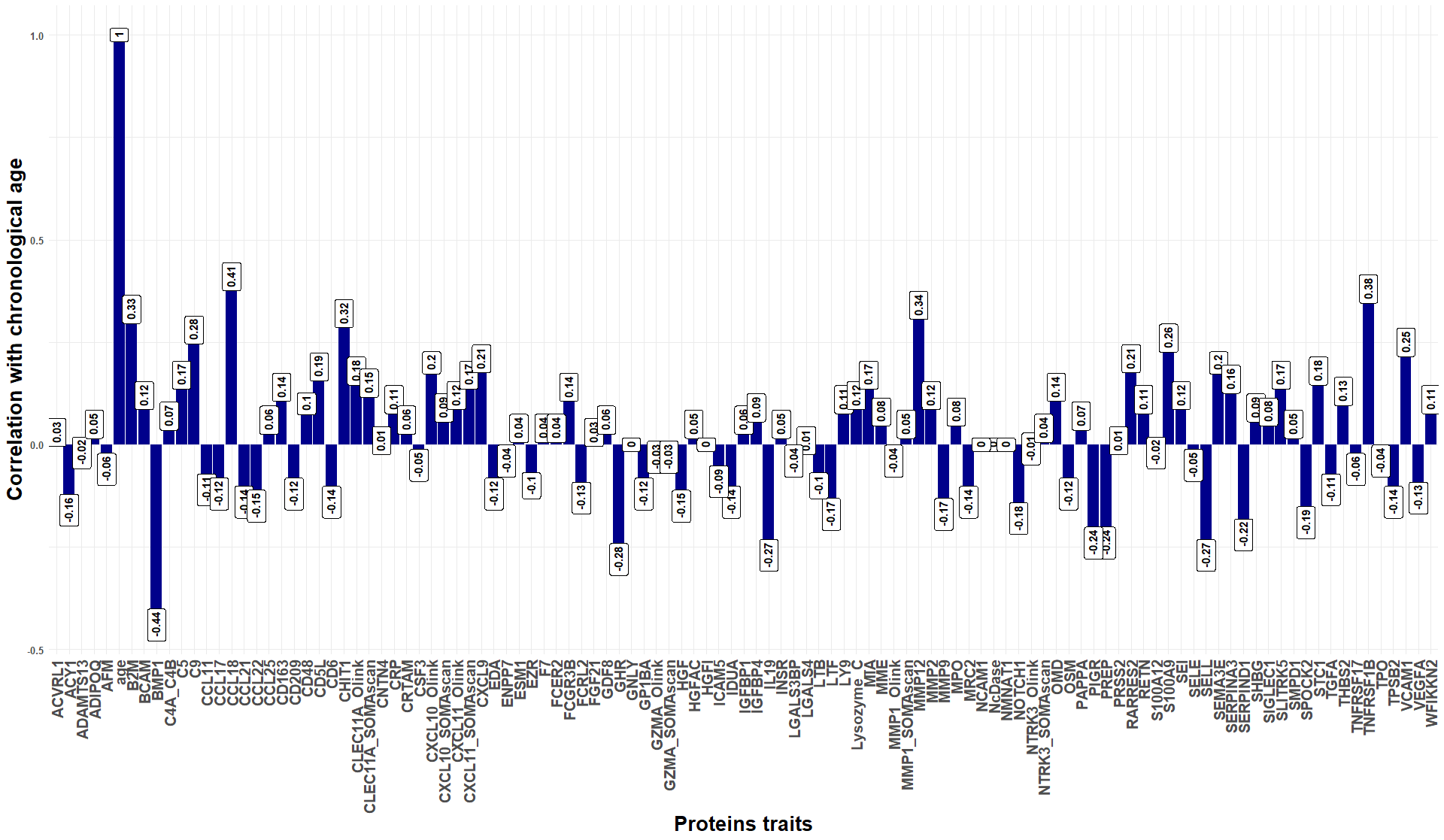


Note: See Table 1 in the manuscript for the 109 protein EpiScores abbreviations.

Supplementary Fig. S3- Strongest positive Pearson correlation plots of the protein EpiScores over age in the Swedish Adoption/Twin Study of Aging - SATSA (n= 374 individuals).


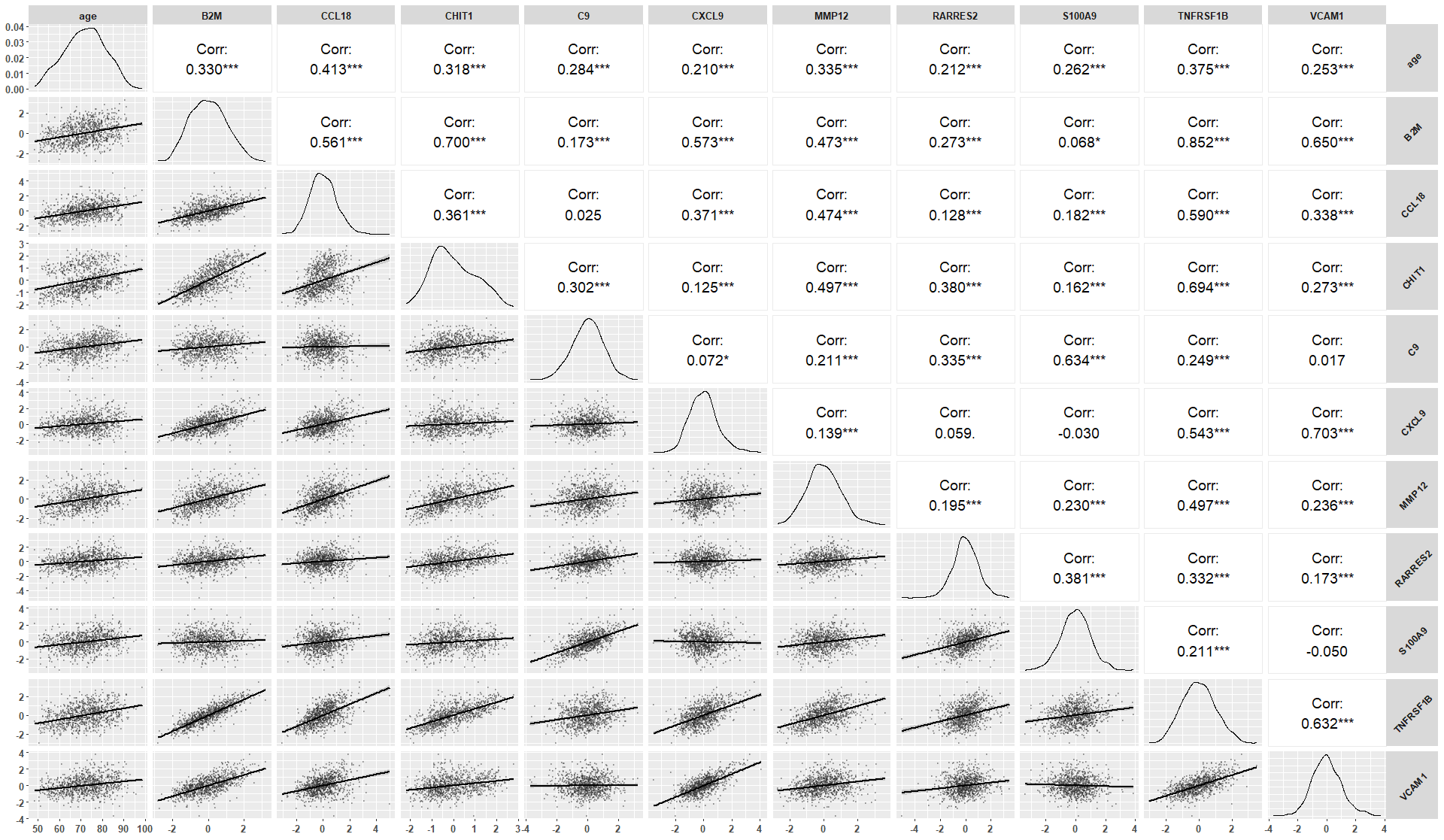


Note: See Table 1 in the manuscript for the 109 protein EpiScores abbreviations.

Supplementary Fig. S4- Strongest negative Pearson correlation plots of the protein EpiScores over age in the Swedish Adoption/Twin Study of Aging - SATSA (n= 374 individuals).


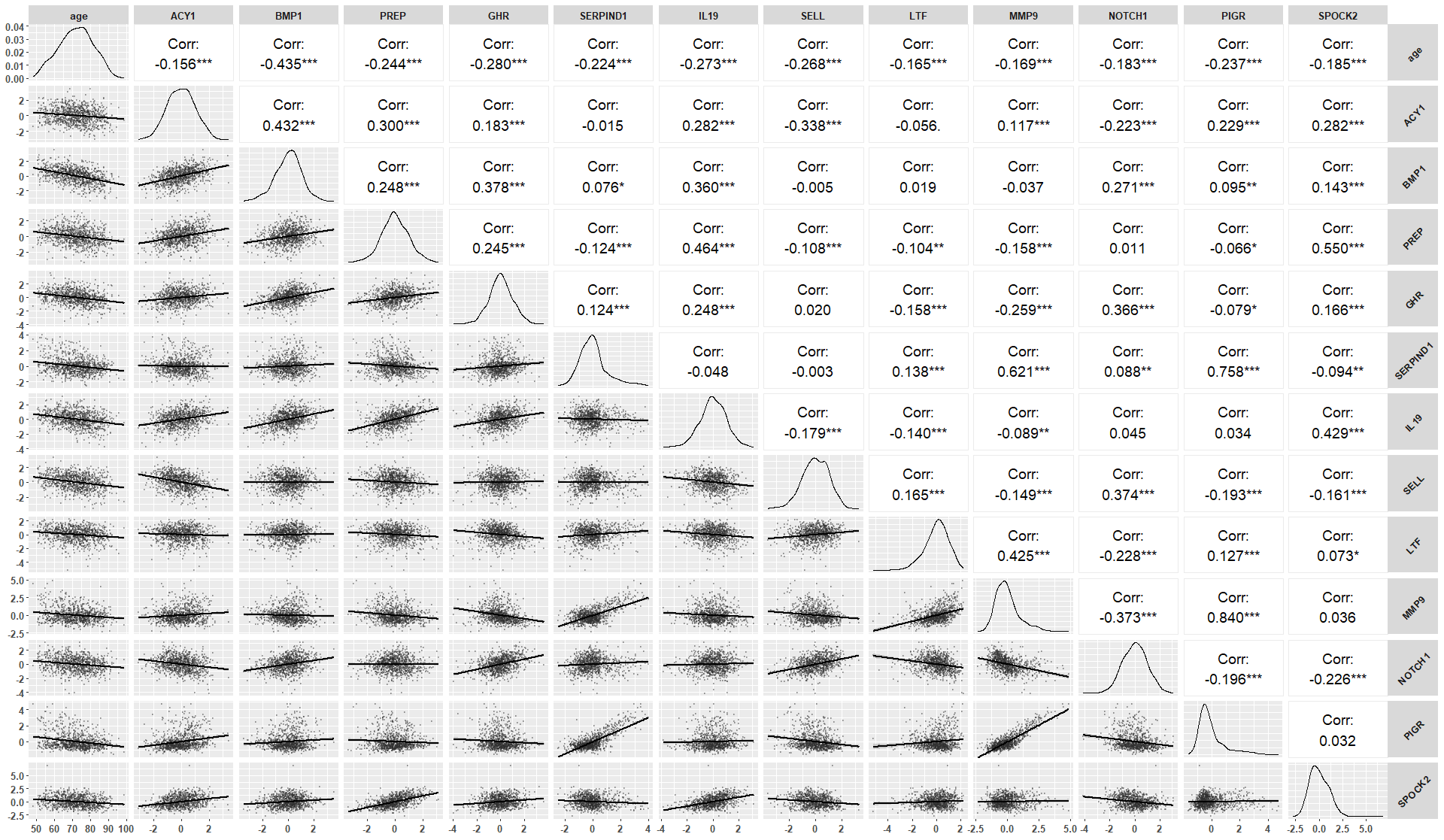


Note: See Table 1 in the manuscript for the 109 protein EpiScores abbreviations.

Supplementary Fig. S5 – Accumulated risk of overall mortality for the risk factor protein EpiScores during the entire study period.


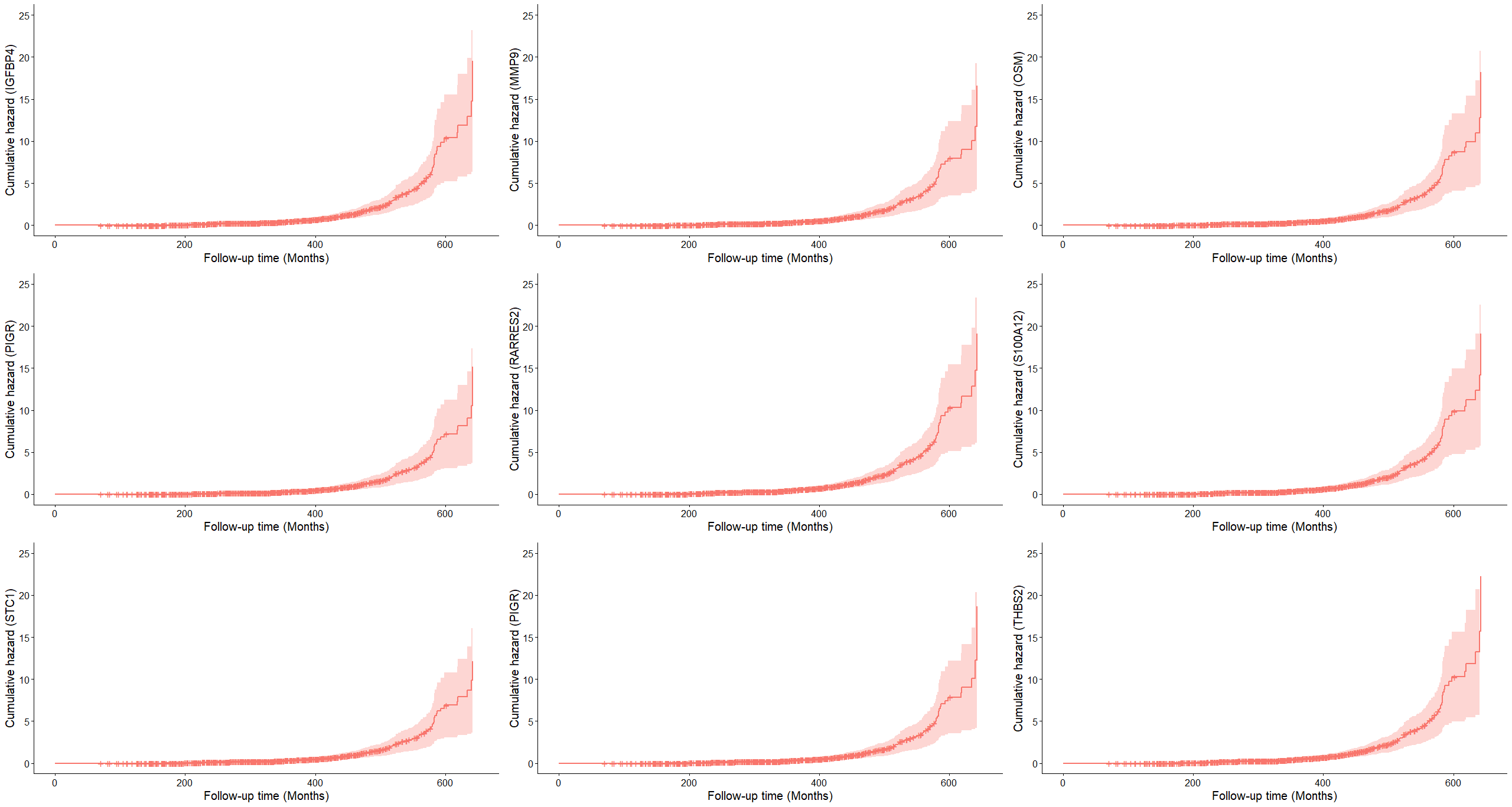


Notes: All models were adjusted for age (time scale), sex, education, body mass index, smoking, and occupation. The cumulative hazards are higher towards the end of the study period. IGFBP4: Insulin-like growth factor-binding protein 4, MMP9: Matrix metalloproteinase 9, OSM: Oncostatin-M, PIGR: Polymeric immunoglobulin receptor, RARRES2: Retinoic acid receptor responder protein 2, S100A12: S100 calcium binding protein A12, STC1: Stanniocalcin 1, TGFA: Transforming growth factor alpha, and THBS2: Thrombospondin 2.

Supplementary Fig. S6 – Accumulated risk of overall mortality for the protective factor protein EpiScores during the entire study period.


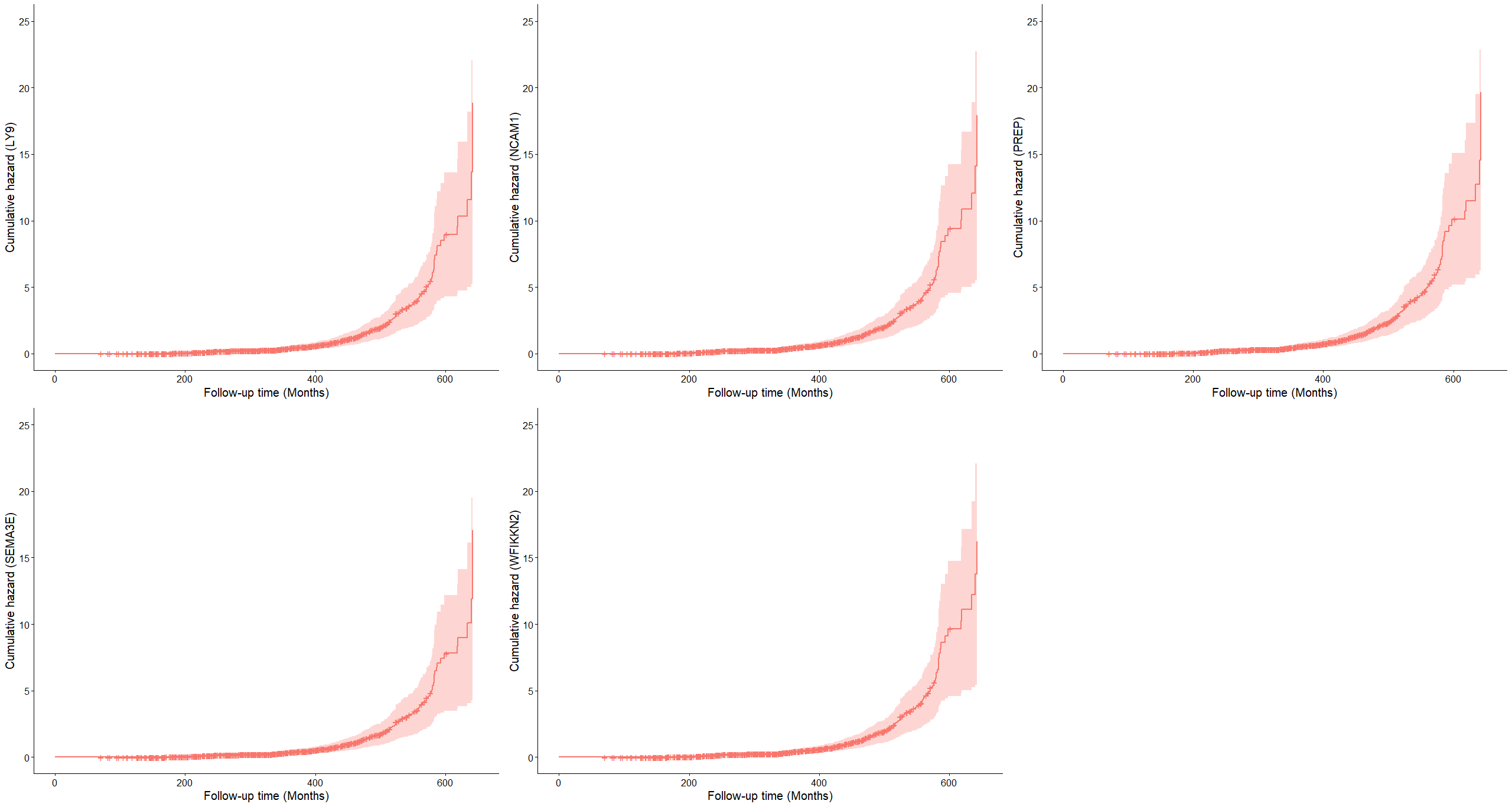


Notes: All models were adjusted for age (time scale), sex, education, body mass index, smoking, and occupation. The cumulative hazards are higher towards the end of the study period. LY9: Lymphocyte antigen 9, NCAM1: Neural cell adhesion molecule 1, PREP: Prolyl endopeptidase, SEMA3E: Semaphorin 3E, and WFIKKN2: WAP, Kazal, immunoglobulin, Kunitz and NTR domain-containing protein 2.

Supplementary Fig. S7 - Correlations between the risk factor protein EpiScores - Swedish Adoption/Twin Study of Aging - SATSA.


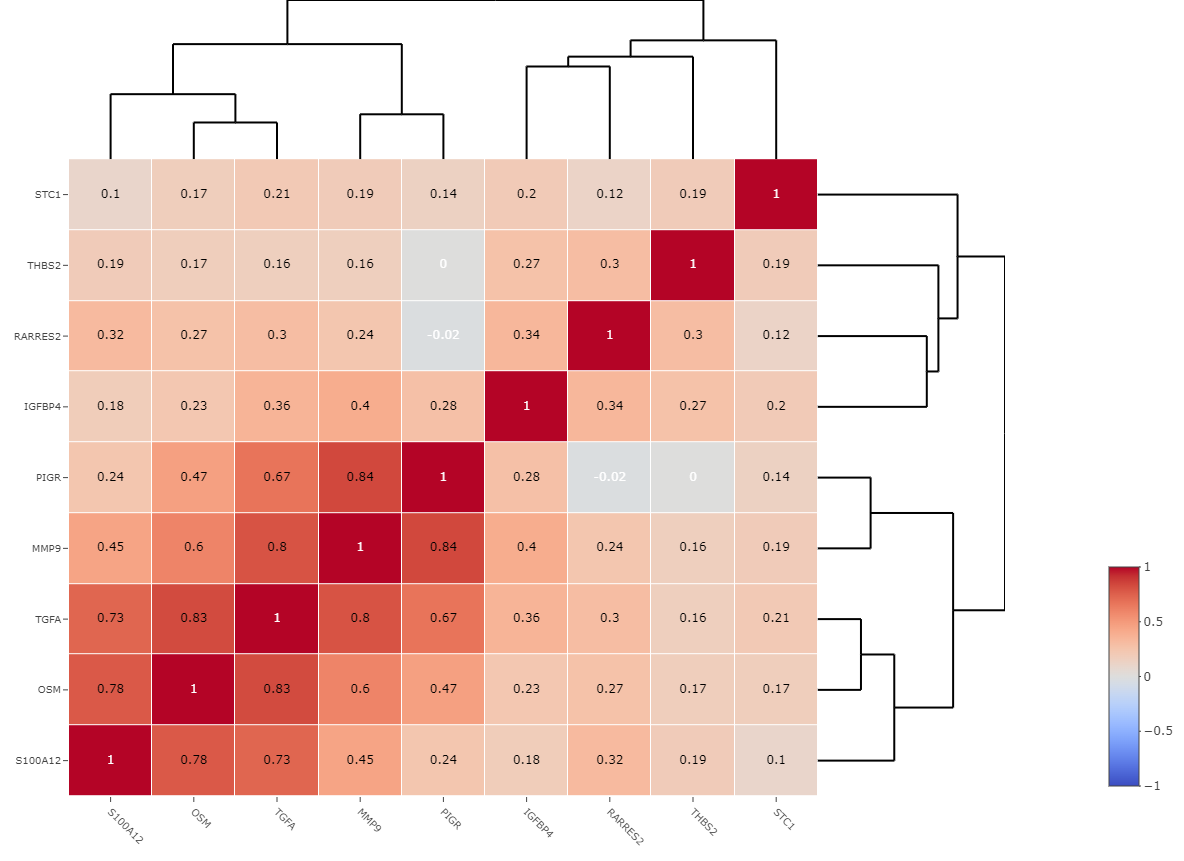


Note: IGFBP4: Insulin-like growth factor-binding protein 4, MMP9: Matrix metalloproteinase 9, OSM: Oncostatin-M, PIGR: Polymeric immunoglobulin receptor, RARRES2: Retinoic acid receptor responder protein 2, S100A12: S100 calcium binding protein A12, STC1: Stanniocalcin 1, TGFA: Transforming growth factor alpha, THBS2: Thrombospondin 2.

Supplementary Fig. S8 - Correlations between the protective factor protein EpiScores - Swedish Adoption/Twin Study of Aging - SATSA.


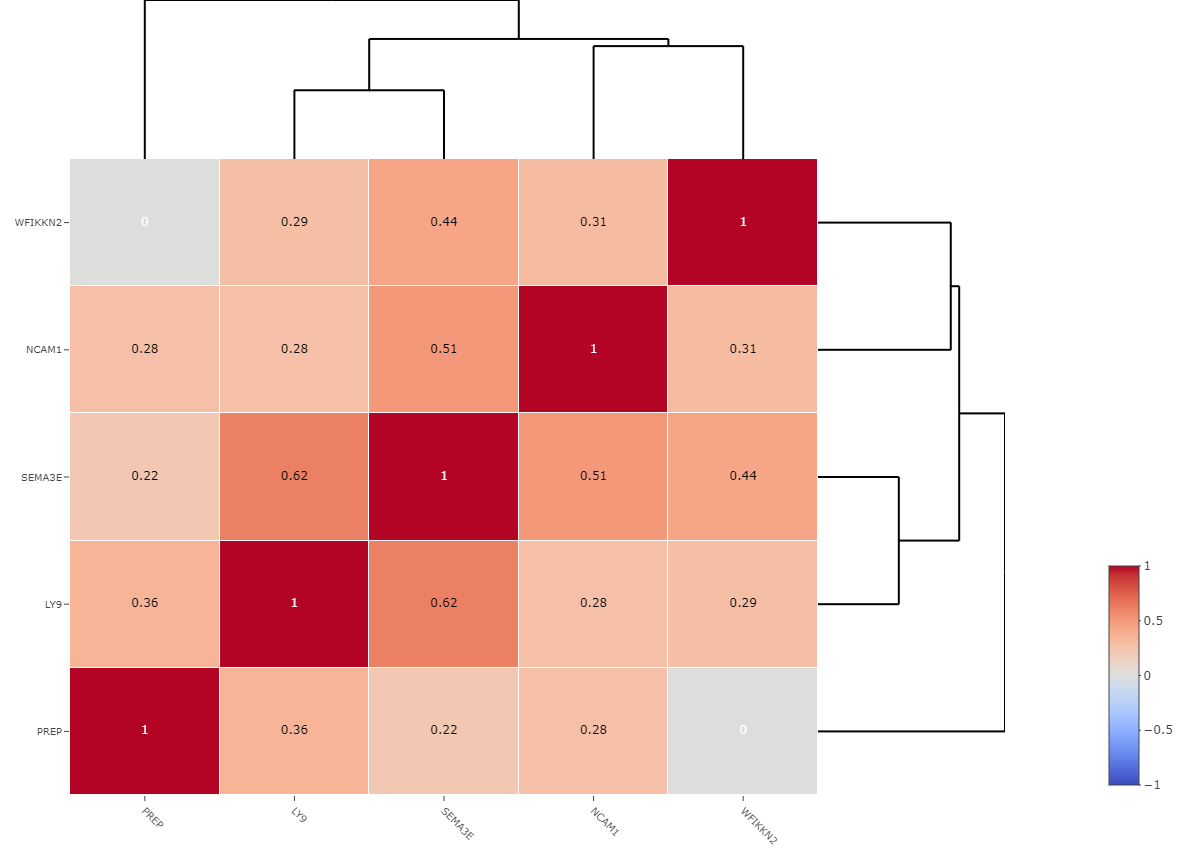


Note: LY9: Lymphocyte antigen 9, NCAM1: Neural cell adhesion molecule 1, PREP: Prolyl endopeptidase, SEMA3E: Semaphorin 3E, WFIKKN2: WAP, Kazal, immunoglobulin, Kunitz and NTR domain-containing protein 2.

Supplementary Table S2 - Descriptives of the 109 protein EpiScores in the first assessment and all study period, the Swedish Adoption/Twin Study of Aging - SATSA (n=374).

| **Protein EpiScores - Median (Min, Max)** | | | |
| --- | --- | --- | --- |
|  | Baseline (n= 374) | All period (all measurements, n=947) |  |
| ADAMTS13 | 0.214 (-3.48, 2.28) | 0.252 (-3.69, 2.76) |  |
| ADIPOQ | 0.122 (-2.71, 2.37) | 0.0803 (-2.71, 2.37) |  |
| AFM | -0.107 (-2.81, 3.75) | -0.0729 (-2.81, 4.03) |  |
| IDUA | -0.0138 (-3.25, 2.24) | 0.0870 (-4.45, 2.61) |  |
| ACY1 | 0.0109 (-2.53, 3.44) | -0.0198 (-3.07, 3.44) |  |
| B2M | -0.0948 (-2.79, 2.58) | -0.0391 (-2.79, 3.22) |  |
| BCAM | 0.576 (-1.73, 0.576) | 0.576 (-1.73, 0.576) |  |
| BMP1 | 0.198 (-2.51, 2.87) | 0.0560 (-3.46, 3.53) |  |
| CCL11 | -0.0978 (-2.15, 3.51) | -0.104 (-2.43, 4.15) |  |
| CCL17 | 0.0523 (-2.60, 3.96) | -0.103 (-2.60, 3.96) |  |
| CCL18 | -0.169 (-3.08, 3.26) | -0.0363 (-3.08, 5.02) |  |
| CCL21 | -0.0605 (-2.63, 2.67) | -0.0657 (-2.80, 3.26) |  |
| CCL22 | -0.00422 (-2.16, 3.98) | -0.0447 (-3.04, 3.98) |  |
| CCL25 | -0.0492 (-2.66, 3.58) | -0.0697 (-2.66, 3.87) |  |
| CD163 | -0.0728 (-2.94, 3.43) | -0.0264 (-3.23, 3.43) |  |
| CD209 | 0.0886 (-2.54, 2.77) | 0.0757 (-2.77, 2.77) |  |
| CD48 | -0.0429 (-2.33, 2.83) | -0.0345 (-2.81, 2.88) |  |
| CD6 | -0.0879 (-2.41, 3.36) | -0.0234 (-3.11, 3.57) |  |
| CD5L | -0.0823 (-2.97, 2.64) | -0.00610 (-2.97, 3.76) |  |
| CHIT1 | -0.267 (-2.18, 2.40) | -0.144 (-2.19, 2.80) |  |
| CLEC11A_Olink | -0.142 (-2.24, 2.93) | -0.0440 (-3.73, 5.84) |  |
| CLEC11A_SOMAscan | -0.123 (-2.72, 2.69) | -0.0557 (-3.43, 4.55) |  |
| F7 | 0.285 (-3.74, 2.34) | 0.287 (-3.74, 2.34) |  |
| C4A_C4B | 0.137 (-4.10, 2.20) | 0.188 (-4.28, 2.20) |  |
| C5 | -0.140 (-3.04, 3.01) | 0.0226 (-5.07, 3.01) |  |
| C9 | -0.0482 (-3.39, 2.96) | 0.0224 (-3.73, 3.25) |  |
| CNTN4 | 0.157 (-3.66, 2.64) | 0.116 (-3.74, 2.64) |  |
| CRP | -0.0471 (-3.21, 3.16) | -0.0437 (-3.21, 3.16) |  |
| CRTAM | -0.146 (-2.30, 3.06) | -0.0806 (-3.09, 4.22) |  |
| CXCL10_Olink | -0.0615 (-3.05, 3.30) | -0.0220 (-3.05, 3.67) |  |
| CXCL10_SOMAscan | -0.222 (-2.16, 2.79) | -0.203 (-2.41, 2.79) |  |
| CXCL11_Olink | -0.0598 (-2.58, 3.05) | -0.0454 (-3.08, 3.46) |  |
| CXCL11_SOMAscan | -0.129 (-2.36, 2.99) | -0.0433 (-2.89, 3.17) |  |
| CXCL9 | -0.126 (-2.62, 4.13) | -0.0404 (-3.53, 4.13) |  |
| SELE | -0.0570 (-3.18, 3.00) | -0.0405 (-3.18, 3.00) |  |
| EDA | -0.215 (-2.14, 3.47) | -0.243 (-2.17, 3.47) |  |
| S100A12 | -0.0909 (-3.36, 3.63) | -0.0419 (-3.37, 3.85) |  |
| ENPP7 | 0.0226 (-1.92, 2.48) | 0.0864 (-2.18, 2.69) |  |
| ESM1 | -0.255 (-1.99, 2.81) | -0.249 (-2.49, 2.81) |  |
| EZR | -0.00753 (-2.71, 3.36) | -0.0555 (-3.33, 4.12) |  |
| FCER2 | -0.0475 (-2.40, 2.95) | -0.0566 (-2.83, 3.61) |  |
| FCGR3B | -0.0673 (-2.66, 2.96) | -0.0362 (-2.86, 3.02) |  |
| FCRL2 | -0.110 (-2.55, 7.22) | -0.168 (-2.55, 7.22) |  |
| FGF21 | -0.0749 (-2.43, 2.98) | -0.0843 (-2.43, 3.12) |  |
| CSF3 | 0.00952 (-4.21, 2.29) | 0.0502 (-4.21, 4.06) |  |
| LGALS4 | -0.178 (-2.51, 4.62) | -0.195 (-2.51, 4.62) |  |
| GDF8 | 0.145 (-3.28, 2.16) | 0.165 (-3.28, 2.69) |  |
| GHR | 0.00305 (-3.51, 3.60) | -0.00359 (-3.94, 3.60) |  |
| GP1BA | -0.0305 (-3.00, 2.75) | -0.146 (-3.00, 3.53) |  |
| GNLY | -0.129 (-2.42, 3.86) | -0.0563 (-2.75, 4.38) |  |
| GZMA_Olink | -0.186 (-2.38, 3.87) | -0.102 (-2.67, 4.35) |  |
| GZMA_SOMAscan | -0.144 (-2.49, 3.93) | -0.0840 (-2.49, 4.37) |  |
| SERPIND1 | -0.0849 (-2.49, 4.01) | -0.115 (-2.49, 4.01) |  |
| HGF | -0.0964 (-2.46, 4.20) | -0.0714 (-2.71, 4.20) |  |
| HGFAC | 0.0550 (-8.55, 1.72) | 0.156 (-8.55, 1.79) |  |
| HGFI | -0.114 (-2.29, 1.56) | -0.0233 (-2.29, 1.69) |  |
| ICAM5 | -0.0477 (-3.61, 3.07) | -0.0692 (-4.21, 3.07) |  |
| IGFBP1 | 0.0869 (-3.03, 3.46) | 0.0422 (-3.03, 4.22) |  |
| IGFBP4 | -0.0586 (-3.88, 5.05) | -0.0226 (-3.88, 5.69) |  |
| INSR | -0.0904 (-2.44, 2.66) | -0.0318 (-2.87, 3.91) |  |
| IL19 | 0.113 (-2.83, 3.13) | -0.000453 (-3.81, 3.13) |  |
| SELL | 0.0788 (-3.59, 3.29) | 0.0307 (-3.59, 3.29) |  |
| LTF | 0.0804 (-5.20, 2.12) | 0.0982 (-5.20, 2.24) |  |
| LGALS3BP | -0.163 (-3.29, 3.58) | -0.0823 (-3.88, 3.58) |  |
| LY9 | 0.00227 (-3.26, 3.47) | -0.0488 (-4.21, 3.47) |  |
| LTB | 0.100 (-2.59, 2.47) | 0.0292 (-2.70, 2.61) |  |
| Lysozyme_C | 0.576 (-1.73, 0.576) | 0.576 (-1.73, 0.576) |  |
| MIA | 0.0185 (-2.68, 2.99) | 0.00489 (-2.68, 2.99) |  |
| MMP1_SOMAscan | -0.121 (-3.11, 3.16) | -0.0961 (-3.11, 3.23) |  |
| MMP12 | -0.174 (-2.69, 3.63) | -0.0444 (-2.69, 3.63) |  |
| MMP2 | 0.576 (-1.73, 0.576) | 0.576 (-1.73, 0.576) |  |
| MMP9 | -0.149 (-2.05, 4.48) | -0.167 (-2.32, 4.85) |  |
| MMP1_Olink | -0.285 (-2.20, 3.11) | -0.206 (-2.32, 3.11) |  |
| MRC2 | 0.0495 (-2.37, 2.45) | -0.0139 (-3.31, 2.86) |  |
| MPO | 0.00582 (-3.57, 3.09) | -0.0146 (-3.57, 3.09) |  |
| NcDase | 0.0976 (-3.61, 2.30) | 0.0897 (-3.93, 2.30) |  |
| NCAM1 | 0.0962 (-2.94, 2.54) | 0.0534 (-3.06, 4.40) |  |
| MME | -0.168 (-2.42, 4.52) | -0.115 (-2.55, 4.52) |  |
| NMNAT1 | -0.0140 (-2.91, 3.79) | -0.0797 (-3.70, 3.79) |  |
| NOTCH1 | 0.0784 (-4.06, 3.11) | 0.00869 (-4.13, 3.11) |  |
| NTRK3_Olink | 0.112 (-4.24, 2.96) | 0.122 (-4.24, 3.14) |  |
| NTRK3_SOMAscan | -0.0545 (-3.44, 3.31) | 0.00632 (-3.44, 3.31) |  |
| OSM | -0.0673 (-3.96, 3.62) | -0.0349 (-3.96, 3.72) |  |
| OMD | 0.0129 (-4.20, 2.28) | 0.0965 (-4.48, 2.33) |  |
| PAPPA | -0.0607 (-2.89, 3.65) | -0.00650 (-3.17, 3.65) |  |
| PIGR | -0.240 (-1.50, 4.73) | -0.299 (-1.65, 4.73) |  |
| PREP | 0.00470 (-3.37, 3.17) | -0.0476 (-3.37, 3.17) |  |
| RARRES2 | -0.0986 (-2.53, 2.87) | -0.0175 (-5.02, 3.45) |  |
| RETN | -0.0730 (-2.71, 2.22) | -0.0335 (-2.71, 2.82) |  |
| S100A9 | -0.0714 (-2.97, 3.84) | 0.0190 (-3.36, 3.87) |  |
| SEMA3E | 0.204 (-4.52, 2.54) | 0.230 (-4.58, 2.54) |  |
| SERPINA3 | 0.0428 (-3.32, 3.05) | 0.0571 (-3.32, 3.05) |  |
| SHBG | 0.0632 (-2.53, 2.53) | 0.0279 (-2.53, 3.00) |  |
| SIGLEC1 | -0.0817 (-2.18, 4.48) | -0.0596 (-2.40, 4.48) |  |
| ACVRL1 | 0.118 (-2.76, 3.67) | 0.145 (-2.76, 3.67) |  |
| SLITRK5 | 0.114 (-4.15, 2.18) | 0.147 (-4.90, 2.55) |  |
| SMPD1 | 0.171 (-4.59, 1.47) | 0.204 (-4.72, 1.70) |  |
| STC1 | -0.238 (-3.04, 2.49) | -0.103 (-3.04, 2.80) |  |
| SPOCK2 | -0.0275 (-2.76, 6.89) | -0.0818 (-2.76, 6.89) |  |
| TGFA | -0.200 (-2.34, 5.06) | -0.146 (-2.79, 5.13) |  |
| THBS2 | -0.0754 (-2.26, 3.41) | -0.0336 (-2.67, 3.41) |  |
| TNFRSF17 | -0.0290 (-2.98, 3.37) | -0.0327 (-3.41, 4.96) |  |
| TNFRSF1B | -0.126 (-2.98, 2.47) | -0.0350 (-2.98, 3.54) |  |
| TPO | -0.0875 (-2.73, 2.73) | -0.128 (-2.73, 3.21) |  |
| PRSS2 | -0.126 (-2.07, 6.13) | -0.116 (-2.62, 6.67) |  |
| TPSB2 | -0.00114 (-2.67, 2.85) | -0.0214 (-3.27, 3.40) |  |
| VCAM1 | -0.187 (-2.71, 2.67) | -0.0310 (-3.68, 3.74) |  |
| VEGFA | -0.0698 (-3.28, 3.41) | -0.0129 (-3.28, 3.41) |  |
| WFIKKN2 | 0.0520 (-4.19, 3.20) | -0.0125 (-4.19, 3.20) |  |

Note: See Table 1 in the manuscript for the 109 protein EpiScores abbreviations.

Supplementary Table S3 - Crude and adjusted hazard ratios between 109 protein EpiScores and overall mortality in the Swedish Adoption/Twin Study of Aging - SATSA (n= 374 individuals).

| **Model 1** | | | **Model 2** | | |
| --- | --- | --- | --- | --- | --- |
| Protein EpiScores | Hazard ratios | p-value | Protein EpiScores | Hazard ratios | FDR  p-value |
| ACVRL1 | 1.20 | 0.02 | ACVRL1 | 1.20 | 0.11 |
| ACY1 | 1.22 | 0.01 | ACY1 | 1.21 | 0.09 |
| ADAMTS13 | 0.91 | 0.13 | ADAMTS13 | 0.96 | 0.75 |
| ADIPOQ | 0.91 | 0.32 | ADIPOQ | 0.85 | 0.31 |
| AFM | 1.00 | 0.97 | AFM | 0.99 | 0.95 |
| B2M | 0.98 | 0.73 | B2M | 0.93 | 0.51 |
| BCAM | 0.99 | 0.90 | BCAM | 1.04 | 0.75 |
| BMP1 | 0.93 | 0.36 | BMP1 | 0.91 | 0.45 |
| C4A_C4B | 1.00 | 0.96 | C4A_C4B | 1.03 | 0.75 |
| C5 | 1.10 | 0.12 | C5 | 1.07 | 0.55 |
| C9 | 1.14 | 0.06 | C9 | 1.11 | 0.35 |
| CCL11 | 1.27 | 0.01 | CCL11 | 1.19 | 0.18 |
| CCL17 | 1.13 | 0.04 | CCL17 | 1.04 | 0.76 |
| CCL18 | 1.03 | 0.65 | CCL18 | 1.01 | 0.90 |
| CCL21 | 0.96 | 0.54 | CCL21 | 0.89 | 0.32 |
| CCL22 | 1.24 | 0.00 | CCL22 | 1.15 | 0.20 |
| CCL25 | 1.00 | 0.94 | CCL25 | 1.00 | 0.95 |
| CD163 | 0.92 | 0.21 | CD163 | 0.87 | 0.16 |
| CD209 | 0.93 | 0.29 | CD209 | 1.01 | 0.95 |
| CD48 | 0.94 | 0.35 | CD48 | 0.88 | 0.24 |
| CD5L | 0.97 | 0.69 | CD5L | 0.92 | 0.45 |
| CD6 | 0.90 | 0.13 | CD6 | 0.91 | 0.43 |
| CHIT1 | 1.02 | 0.76 | CHIT1 | 0.97 | 0.82 |
| CLEC11A_Olink | 0.98 | 0.74 | CLEC11A_Olink | 1.02 | 0.89 |
| CLEC11A_SOMAscan | 0.97 | 0.67 | CLEC11A_SOMAscan | 0.99 | 0.95 |
| CNTN4 | 0.82 | 0.00 | CNTN4 | 0.86 | 0.13 |
| CRP | 1.18 | 0.02 | CRP | 1.14 | 0.20 |
| CRTAM | 0.96 | 0.50 | CRTAM | 0.95 | 0.66 |
| CSF3 | 1.12 | 0.07 | CSF3 | 1.10 | 0.35 |
| CXCL10_Olink | 0.95 | 0.41 | CXCL10_Olink | 0.94 | 0.61 |
| CXCL10_SOMAscan | 1.01 | 0.89 | CXCL10_SOMAscan | 0.96 | 0.75 |
| CXCL11_Olink | 0.98 | 0.75 | CXCL11_Olink | 0.96 | 0.75 |
| CXCL11_SOMAscan | 0.96 | 0.52 | CXCL11_SOMAscan | 0.90 | 0.32 |
| CXCL9 | 0.97 | 0.63 | CXCL9 | 0.92 | 0.49 |
| EDA | 0.98 | 0.79 | EDA | 0.94 | 0.61 |
| ENPP7 | 1.05 | 0.48 | ENPP7 | 1.06 | 0.63 |
| ESM1 | 0.95 | 0.47 | ESM1 | 0.94 | 0.63 |
| EZR | 0.96 | 0.59 | EZR | 0.98 | 0.90 |
| F7 | 1.02 | 0.77 | F7 | 0.97 | 0.79 |
| FCER2 | 0.91 | 0.12 | FCER2 | 0.92 | 0.45 |
| FCGR3B | 0.93 | 0.29 | FCGR3B | 0.88 | 0.21 |
| FCRL2 | 0.96 | 0.53 | FCRL2 | 0.89 | 0.32 |
| FGF21 | 1.17 | 0.01 | FGF21 | 1.14 | 0.15 |
| GDF8 | 0.93 | 0.28 | GDF8 | 1.02 | 0.89 |
| GHR | 1.04 | 0.47 | GHR | 1.05 | 0.67 |
| GNLY | 1.01 | 0.89 | GNLY | 0.98 | 0.89 |
| GP1BA | 0.93 | 0.28 | GP1BA | 0.92 | 0.40 |
| GZMA_Olink | 0.98 | 0.74 | GZMA_Olink | 0.94 | 0.63 |
| GZMA_SOMAscan | 0.94 | 0.33 | GZMA_SOMAscan | 0.94 | 0.63 |
| HGF | 1.31 | 0.00 | HGF | 1.24 | 0.06 |
| HGFAC | 0.95 | 0.40 | HGFAC | 0.99 | 0.93 |
| HGFI | 0.86 | 0.01 | HGFI | 0.86 | 0.10 |
| ICAM5 | 1.17 | 0.01 | ICAM5 | 1.07 | 0.55 |
| IDUA | 1.01 | 0.89 | IDUA | 0.98 | 0.88 |
| IGFBP1 | 0.89 | 0.07 | IGFBP1 | 0.88 | 0.20 |
| IGFBP4 | 1.25 | 0.00 | IGFBP4 | 1.23 | 0.03 |
| IL19 | 0.98 | 0.71 | IL19 | 0.96 | 0.74 |
| INSR | 0.90 | 0.11 | INSR | 0.90 | 0.32 |
| LGALS3BP | 1.13 | 0.06 | LGALS3BP | 1.11 | 0.32 |
| LGALS4 | 1.11 | 0.12 | LGALS4 | 1.10 | 0.32 |
| LTB | 0.79 | 0.00 | LTB | 0.86 | 0.24 |
| LTF | 1.03 | 0.67 | LTF | 0.99 | 0.93 |
| LY9 | 0.80 | 0.00 | LY9 | 0.80 | 0.03 |
| Lysozyme_C | 0.99 | 0.90 | Lysozyme_C | 1.04 | 0.75 |
| MIA | 0.91 | 0.16 | MIA | 0.93 | 0.53 |
| MME | 0.98 | 0.69 | MME | 1.00 | 0.96 |
| MMP1_Olink | 1.08 | 0.23 | MMP1_Olink | 1.02 | 0.88 |
| MMP1_SOMAscan | 1.06 | 0.36 | MMP1_SOMAscan | 0.98 | 0.82 |
| MMP12 | 1.27 | 0.00 | MMP12 | 1.17 | 0.13 |
| MMP2 | 0.99 | 0.90 | MMP2 | 1.04 | 0.75 |
| MMP9 | 1.34 | 0.00 | MMP9 | 1.24 | 0.05 |
| MPO | 1.12 | 0.07 | MPO | 1.09 | 0.43 |
| MRC2 | 0.87 | 0.03 | MRC2 | 0.89 | 0.27 |
| NCAM1 | 0.77 | 0.00 | NCAM1 | 0.78 | 0.03 |
| NcDase | 0.93 | 0.28 | NcDase | 0.95 | 0.68 |
| NMNAT1 | 0.96 | 0.48 | NMNAT1 | 0.97 | 0.79 |
| NOTCH1 | 0.93 | 0.24 | NOTCH1 | 0.96 | 0.75 |
| NTRK3_Olink | 0.73 | 0.00 | NTRK3_Olink | 0.79 | 0.04 |
| NTRK3_SOMAscan | 0.83 | 0.01 | NTRK3_SOMAscan | 0.89 | 0.24 |
| OMD | 0.83 | 0.01 | OMD | 0.89 | 0.30 |
| OSM | 1.40 | 0.00 | OSM | 1.32 | 0.00 |
| PAPPA | 1.01 | 0.84 | PAPPA | 0.97 | 0.80 |
| PIGR | 1.41 | 0.00 | PIGR | 1.31 | 0.03 |
| PREP | 0.86 | 0.02 | PREP | 0.83 | 0.05 |
| PRSS2 | 1.05 | 0.44 | PRSS2 | 1.06 | 0.63 |
| RARRES2 | 1.20 | 0.00 | RARRES2 | 1.22 | 0.03 |
| RETN | 1.00 | 0.96 | RETN | 0.97 | 0.80 |
| S100A12 | 1.24 | 0.00 | S100A12 | 1.22 | 0.03 |
| S100A9 | 1.15 | 0.04 | S100A9 | 1.15 | 0.20 |
| SELE | 1.15 | 0.08 | SELE | 1.21 | 0.10 |
| SELL | 0.91 | 0.14 | SELL | 0.90 | 0.35 |
| SEMA3E | 0.74 | 0.00 | SEMA3E | 0.80 | 0.05 |
| SERPINA3 | 1.01 | 0.92 | SERPINA3 | 1.05 | 0.72 |
| SERPIND1 | 1.35 | 0.00 | SERPIND1 | 1.22 | 0.06 |
| SHBG | 0.97 | 0.71 | SHBG | 0.91 | 0.46 |
| SIGLEC1 | 0.96 | 0.61 | SIGLEC1 | 0.92 | 0.48 |
| SLITRK5 | 0.75 | 0.00 | SLITRK5 | 0.83 | 0.15 |
| SMPD1 | 0.97 | 0.69 | SMPD1 | 0.95 | 0.66 |
| SPOCK2 | 0.89 | 0.10 | SPOCK2 | 0.87 | 0.20 |
| STC1 | 1.43 | 0.00 | STC1 | 1.49 | 0.00 |
| TGFA | 1.40 | 0.00 | TGFA | 1.32 | 0.00 |
| THBS2 | 1.22 | 0.00 | THBS2 | 1.19 | 0.05 |
| TNFRSF17 | 0.99 | 0.81 | TNFRSF17 | 0.96 | 0.75 |
| TNFRSF1B | 1.06 | 0.41 | TNFRSF1B | 1.02 | 0.85 |
| TPO | 0.97 | 0.67 | TPO | 0.94 | 0.56 |
| TPSB2 | 1.09 | 0.18 | TPSB2 | 1.07 | 0.53 |
| VCAM1 | 0.95 | 0.45 | VCAM1 | 0.91 | 0.32 |
| VEGFA | 1.16 | 0.04 | VEGFA | 1.11 | 0.35 |
| WFIKKN2 | 0.76 | 0.00 | WFIKKN2 | 0.80 | 0.03 |

Note: Model 1 were adjusted for age (time scale) and sex. Model 2 were adjusted age (time scale), sex, education, smoking, body mass index, and occupation. See Table 1 in the manuscript for the 109 protein EpiScores abbreviations. FDR: False Discovery Rate.

Supplementary Table S4 - Schoenfeld residual analyses from the Cox proportional hazards adjusted models, the Swedish Adoption/Twin Study of Aging - SATSA (n= 374 individuals).

|  | **EpiScore-specific Schoenfeld residual P-value** | **Global Schoenfeld residual P-value** |
| --- | --- | --- |
| **Protein epigenetic scores considered risk factor** |  |  |
| IGFBP4 | 0.19 | 0.42 |
| MMP9 | 0.10 | 0.35 |
| OSM | 0.91 | 0.64 |
| PIGR | 0.37 | 0.44 |
| RARRES2 | 0.11 | 0.34 |
| S100A12 | 0.67 | 0.62 |
| STC1 | 0.30 | 0.53 |
| TGFA | 0.99 | 0.60 |
| THBS2 | 0.70 | 0.57 |
| **Protein epigenetic scores considered protective factor** |  |  |
| LY9 | 0.80 | 0.73 |
| NCAM1 | 0.48 | 0.60 |
| NTRK3 – Olink | **0.02** | 0.14 |
| PREP | 0.50 | 0.69 |
| SEMA3E | 0.81 | 0.66 |
| WFIKKN2 | 0.53 | 0.65 |

Note: Bold values represent the protein EpiScores that violated the assumption of proportional hazards (p-value ≤0.05). The models were adjusted age (time scale), sex, education, smoking, body mass index, and occupation. IGFBP4: Insulin-like growth factor-binding protein 4, LY9: Lymphocyte antigen 9, MMP9: Matrix metalloproteinase 9, NCAM1: Neural cell adhesion molecule 1, OSM: Oncostatin-M, PIGR: Polymeric immunoglobulin receptor, PREP: Prolyl endopeptidase, RARRES2: Retinoic acid receptor responder protein 2, S100A12: S100 calcium binding protein A12, SEMA3E: Semaphorin 3E, STC1: Stanniocalcin 1, TGFA: Transforming growth factor alpha, THBS2: Thrombospondin 2, WFIKKN2: WAP, Kazal, immunoglobulin, Kunitz and NTR domain-containing protein 2.

Supplementary Fig. S9 – Schoenfeld residuals plot for NTRK3 - Olink protein EpiScore, the Swedish Adoption/Twin Study of Aging - SATSA.


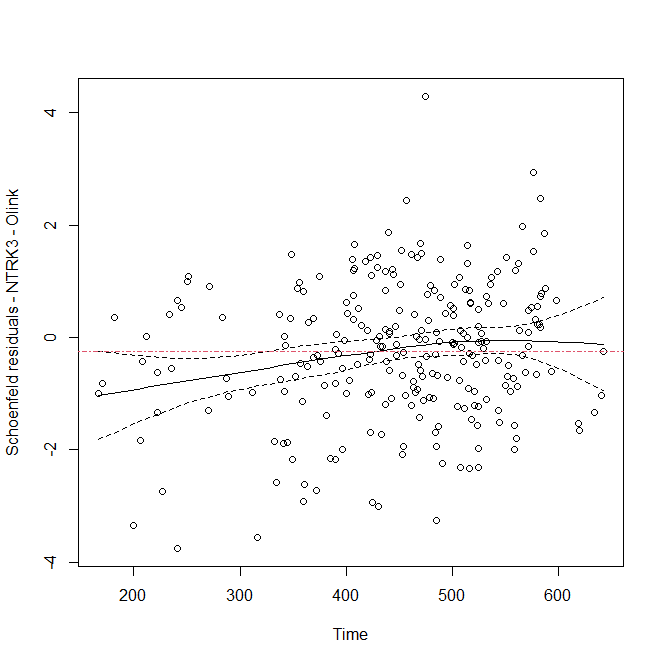


Supplementary Table S5 – Descriptive of deviance residuals from the Cox proportional hazards adjusted models, the Swedish Adoption/Twin Study of Aging - SATSA (n= 491 individuals).

|  | **Minimum** | **Median** | **Maximum** |
| --- | --- | --- | --- |
| **Protein epigenetic scores - risk factor models** |  |  |  |
| IGFBP4 | -2.20 | -0.28 | 2.78 |
| MMP9 | -2.05 | -0.28 | 2.90 |
| OSM | -2.14 | -0.28 | 2.87 |
| PIGR | -2.15 | -0.28 | 2.84 |
| RARRES2 | -2.19 | -0.28 | 2.94 |
| S100A12 | -2.11 | -0.28 | 2.94 |
| STC1 | -2.16 | -0.28 | 2.84 |
| TGFA | -2.09 | -0.27 | 2.83 |
| THBS2 | -2.20 | -0.29 | 2.83 |
| **Protein epigenetic scores - protective factor models** |  |  |  |
| LY9 | -2.01 | -0.28 | 3.04 |
| NCAM1 | -1.96 | -0.28 | 2.87 |
| NTRK3 - Olink | -2.12 | -0.28 | 2.80 |
| PREP | -2.15 | -0.28 | 3.03 |
| SEMA3E | -2.16 | -0.28 | 2.98 |
| WFIKKN2 | -2.25 | -0.28 | 2.97 |

Note: Values >2 and < -2 are considered outlier. The models were adjusted age (time scale), sex, education, smoking, body mass index, and occupation. IGFBP4: Insulin-like growth factor-binding protein 4, LY9: Lymphocyte antigen 9, MMP9: Matrix metalloproteinase 9, NCAM1: Neural cell adhesion molecule 1, OSM: Oncostatin-M, PIGR: Polymeric immunoglobulin receptor, PREP: Prolyl endopeptidase, RARRES2: Retinoic acid receptor responder protein 2, S100A12: S100 calcium binding protein A12, SEMA3E: Semaphorin 3E, STC1: Stanniocalcin 1, TGFA: Transforming growth factor alpha, THBS2: Thrombospondin 2, WFIKKN2: WAP, Kazal, immunoglobulin, Kunitz and NTR domain-containing protein 2.

Supplementary Table S6 – Descriptive of score residuals from the Cox proportional hazards adjusted models, the Swedish Adoption/Twin Study of Aging - SATSA (n= 491 individuals).

|  | **Minimum** | **Median** | **Maximum** |
| --- | --- | --- | --- |
| **Protein epigenetic scores - risk factor models** |  |  |  |
| IGFBP4 | -0.30 | 0.00 | 0.22 |
| MMP9 | -0.38 | 0.00 | 0.12 |
| OSM | -0.22 | 0.00 | 0.21 |
| PIGR | -0.33 | 0.00 | 0.12 |
| RARRES2 | -0.29 | 0.00 | 0.19 |
| S100A12 | -0.25 | 0.00 | 0.27 |
| STC1 | -0.14 | 0.00 | 0.15 |
| TGFA | -0.40 | 0.00 | 0.21 |
| THBS2 | -0.27 | 0.00 | 0.22 |
| **Protein epigenetic scores - protective factor models** |  |  |  |
| LY9 | -0.20 | -0.00 | 0.22 |
| NCAM1 | -0.23 | -0.00 | 0.22 |
| NTRK3 - Olink | -0.18 | -0.00 | 0.23 |
| PREP | -0.18 | -0.00 | 0.23 |
| SEMA3E | -0.16 | -0.00 | 0.39 |
| WFIKKN2 | -0.19 | -0.00 | 0.26 |

Note: The score residuals help in identifying influential or extreme observations. The values should be around 0. The models were adjusted age (time scale), sex, education, smoking, body mass index, and occupation. IGFBP4: Insulin-like growth factor-binding protein 4, LY9: Lymphocyte antigen 9, MMP9: Matrix metalloproteinase 9, NCAM1: Neural cell adhesion molecule 1, OSM: Oncostatin-M, PIGR: Polymeric immunoglobulin receptor, PREP: Prolyl endopeptidase, RARRES2: Retinoic acid receptor responder protein 2, S100A12: S100 calcium binding protein A12, SEMA3E: Semaphorin 3E, STC1: Stanniocalcin 1, TGFA: Transforming growth factor alpha, THBS2: Thrombospondin 2, WFIKKN2: WAP, Kazal, immunoglobulin, Kunitz and NTR domain-containing protein 2.

Supplementary Table S7 – Cox proportional hazards from co-twin control analyses, the Swedish Adoption/Twin Study of Aging - SATSA (n= 491 individuals).

|  | **Within pair (95% CI)** | **Within monozygotic twin (95% CI)** | **Within dizygotic twin (95% CI)** |
| --- | --- | --- | --- |
| **Protein epigenetic scores - risk factor models** |  |  |  |
| IGFBP4 | 1.27 (0.94-1.72) | **1.91 (1.01-3.58)** | 1.09 (0.74-1.59) |
| MMP9 | **1.54 (1.01-2.33)** | **16.86 (3.03-93.70)** | 1.12 (0.70-1.78) |
| OSM | **1.45 (1.06-1.99)** | **3.09 (1.36-7.02)** | 1.16 (0.80-1.68) |
| PIGR | 1.32 (0.90-1.93) | **4.31 (1.62-11.45)** | 0.88 (0.55-1.41) |
| RARRES2 | **1.42 (1.06-1.89)** | 2.00 (0.99-4.02) | 1.36 (0.97-1.92) |
| S100A12 | **1.35 (1.02-1.78)** | **2.49 (1.21-5.12)** | 1.16 (0.84-1.60) |
| STC1 | 1.46 (0.88-2.42) | 1.62 (0.55-4.79) | 1.43 (0.80-2.55) |
| TGFA | **1.40 (1.01-1.95)** | **4.34 (1.67-11.27)** | 1.09 (0.74-1.59) |
| THBS2 | 1.37 (0.98-1.92) | 2.20 (0.90-5.35) | 1.27 (0.88-1.86) |
| **Protein epigenetic scores - protective factor models** |  |  |  |
| LY9 | 0.91 (0.65-1.26) | 0.69 (0.34-1.38) | 0.93 (0.63-1.38) |
| NCAM1 | 0.79 (0.59-1.08) | 0.72 (0.37-1.40) | 0.78 (0.54-1.11) |
| PREP | 0.87 (0.66-1.15) | 0.64 (0.34-1.22) | 0.92 (0.67-1.26) |
| SEMA3E | 0.78 (0.53-1.14) | **0.26 (0.10-0.68)** | 1.04 (0.65-1.65) |
| WFIKKN2 | 0.75 (0.53-1.05) | 0.70 (0.34-1.44) | 0.77 (0.52-1.12) |

Note: All models were adjusted for age (time scale), education, body mass index, and smoking. All twin pairs were of the same sex. 95% CI: Confidence interval 95%. Bold values represent significant values (95% CI). IGFBP4: Insulin-like growth factor-binding protein 4, LY9: Lymphocyte antigen 9, MMP9: Matrix metalloproteinase 9, NCAM1: Neural cell adhesion molecule 1, OSM: Oncostatin-M, PIGR: Polymeric immunoglobulin receptor, PREP: Prolyl endopeptidase, RARRES2: Retinoic acid receptor responder protein 2, S100A12: S100 calcium binding protein A12, SEMA3E: Semaphorin 3E, STC1: Stanniocalcin 1, TGFA: Transforming growth factor alpha, THBS2: Thrombospondin 2, WFIKKN2: WAP, Kazal, immunoglobulin, Kunitz and NTR domain-containing protein 2.


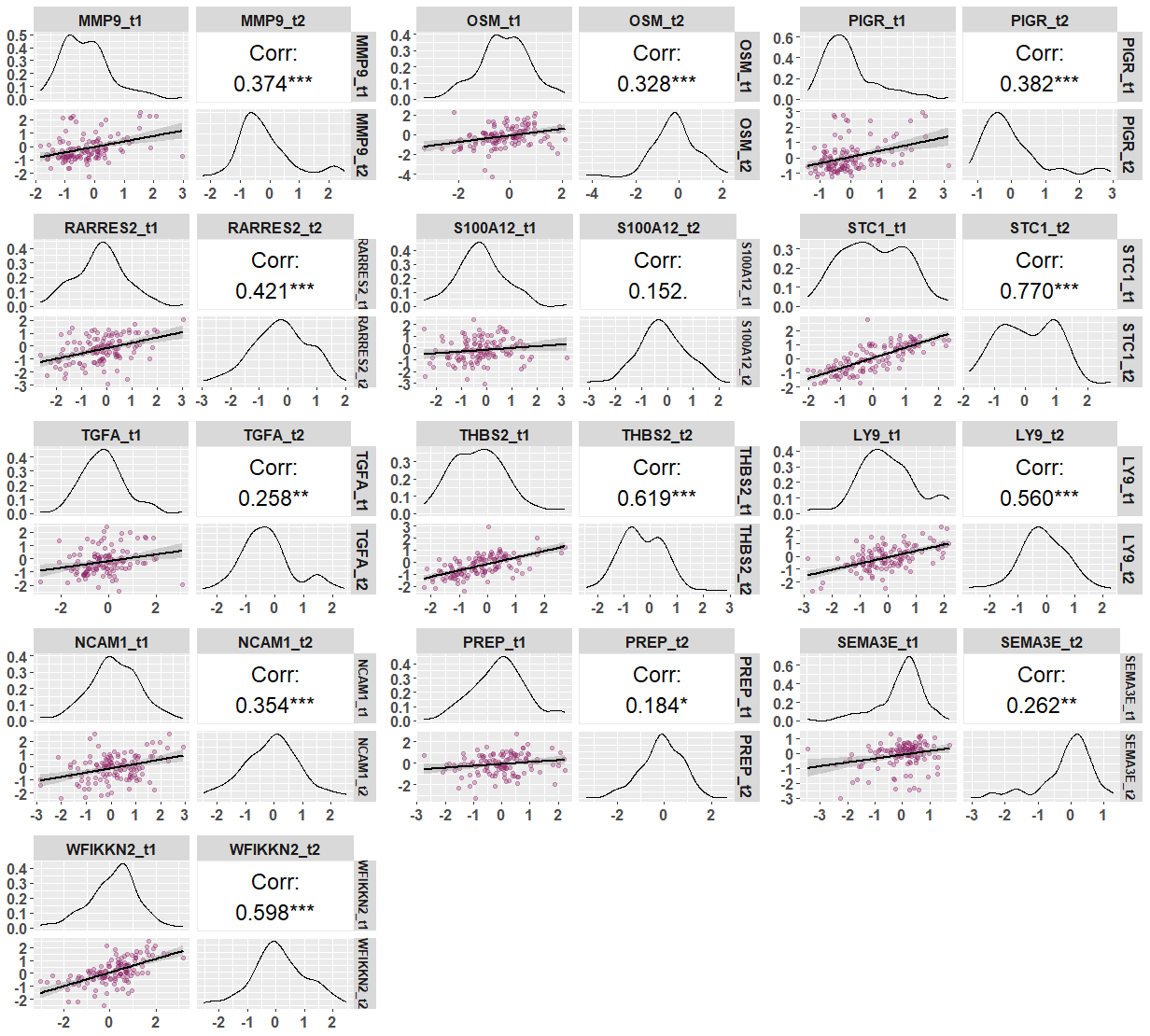
Supplementary Fig. S10 – Protein EpiScores correlation between monozygotic twins, the Swedish Adoption/Twin Study of Aging - SATSA (n= 127 individuals).

Note: IGFBP4: Insulin-like growth factor-binding protein 4, LY9: Lymphocyte antigen 9, MMP9: Matrix metalloproteinase 9, NCAM1: Neural cell adhesion molecule 1, OSM: Oncostatin-M, PIGR: Polymeric immunoglobulin receptor, PREP: Prolyl endopeptidase, RARRES2: Retinoic acid receptor responder protein 2, S100A12: S100 calcium binding protein A12, SEMA3E: Semaphorin 3E, STC1: Stanniocalcin 1, TGFA: Transforming growth factor alpha, THBS2: Thrombospondin 2, WFIKKN2: WAP, Kazal, immunoglobulin, Kunitz and NTR domain-containing protein 2.
